# Animal Density and Track Counts: Understanding the Nature of Observations Based on Animal Movements

**DOI:** 10.1371/journal.pone.0096598

**Published:** 2014-05-28

**Authors:** Derek Keeping, Rick Pelletier

**Affiliations:** Department of Renewable Resources, University of Alberta, Edmonton, Alberta, Canada; University of Illinois at Urbana-Champaign, United States of America

## Abstract

Counting animals to estimate their population sizes is often essential for their management and conservation. Since practitioners frequently rely on indirect observations of animals, it is important to better understand the relationship between such indirect indices and animal abundance. The Formozov-Malyshev-Pereleshin (FMP) formula provides a theoretical foundation for understanding the relationship between animal track counts and the true density of species. Although this analytical method potentially has universal applicability wherever animals are readily detectable by their tracks, it has long been unique to Russia and remains widely underappreciated. In this paper, we provide a test of the FMP formula by isolating the influence of animal travel path tortuosity (i.e., convolutedness) on track counts. We employed simulations using virtual and empirical data, in addition to a field test comparing FMP estimates with independent estimates from line transect distance sampling. We verify that track counts (total intersections between animals and transects) are determined entirely by density and daily movement distances. Hence, the FMP estimator is theoretically robust against potential biases from specific shapes or patterns of animal movement paths if transects are randomly situated with respect to those movements (i.e., the transects do not influence animals’ movements). However, detectability (the detection probability of individual animals) is not determined simply by daily travel distance but also by tortuosity, so ensuring that all intersections with transects are counted regardless of the number of individual animals that made them becomes critical for an accurate density estimate. Additionally, although tortuosity has no bearing on mean track encounter rates, it does affect encounter rate variance and therefore estimate precision. We discuss how these fundamental principles made explicit by the FMP formula have widespread implications for methods of assessing animal abundance that rely on indirect observations.

## Introduction

Estimating animal numbers is often a basic requirement for determining the status of species. However, this task is deceptively simple and no single best approach exists; techniques that work well in some situations are useless in others [1]. Many terrestrial mammals are nocturnal, cryptic in appearance, and generally adept at avoiding being seen, which limits well-developed methods of direct observation, including distance sampling [2]–[5]. These challenges leave indirect observation, for example via animal tracks or remote photography, as often the only realistic option.

In many parts of the world, conservationists rely on animal track surveys as an indispensable tool. Animal track surveys are used in a range of efforts, such as large-scale biodiversity monitoring in northern Europe [6], [7], North America [8], and Australia [9], habitat and land use impact assessments [10]–[16], planning sustainable harvest of ungulates and furbearers [17]–[23], managing invasive species [24]–[27], and monitoring endangered populations such as black rhino *Diceros bicornis* [28], tigers *Panthera tigris* [29], [30], Florida panther *Puma concolor* [31], wolverine *Gulo gulo* [32], [33], and polar bears *Ursus maritimus* [34]. Where substrates are suitable, practitioners continue to use track surveys because they are simple, practical, inexpensive, and readily produce detections for all terrestrial animals including those otherwise difficult to detect. Ironically, science may have origins in tracking. Liebenberg [35] notes that a fully modern human brain evolved when all humans were hunter-gatherers and argues that efficient tracking techniques necessary for successful acquisition of prey still practiced by contemporary hunter-gatherers were the origin of creative hypothetico-deductive thought processes now made explicit by modern science.

In spite of this widespread reliance on tracks and historical perspective, theoretical developments to advance our understanding of the relationship between tracks and their makers’ true population density have generally been sidelined in favour of direct sightings or technologically advanced approaches to wildlife science. While there have been some creative approaches to estimating density from track counts [36], [37], such counts are most often relegated to simple indices of relative abundance (e.g., [38]–[42]). Sometimes, these indices are calibrated to true density through double sampling [43]–[47]. In both cases, the relationship between the index and the population density is assumed to be linear, monotonic, and stable. It is this failure to account for changing detection probabilities that has prompted criticisms on the use of such indices [48], [49], despite urgent practical reasons for conservationists defending their use [50], [51]. Wildlife management and conservation practitioners around the world would benefit from a better understanding of the mechanistic basis linking indirect observations, such as track counts, to animal abundance.

The Formozov-Malyshev-Pereleshin (FMP) formula is an analytical method for converting track counts to population density. This formula was first developed over 80 years ago to estimate game numbers in the snowy regions of Russia. The formula’s conceptual basis and derivation is described in [52]. In short, it is derived from the probabilistic intersection of lines of specified lengths within a defined area and therefore describes the relationship of both transect length and animal day range (lines) to track counts (intersections) and animal density. The formula has the following form:
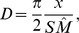
where 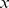 is the total number of track crossings over one 24-hour period, 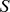 is the total transect length, and 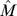 is the mean daily travel distance for all animals in the study area.

Since its recent introduction to the English scientific literature [52], the FMP formula has prompted a closer look at ideal gas models and the development of a parallel approach to estimate density using camera trapping rates [53]. However, despite widespread applicability, the FMP formula still remains underappreciated and is rarely applied outside of Russia. Previous work has addressed the formula’s theoretical basis [52], but perhaps the simplicity of the derived relationship leaves lingering doubts regarding the spatial element of animal movement influencing detectability and encounter rates. Concerns over the non-randomness of animal movements seem to persist (see [54]), although these concerns have been addressed to some extent in recent reviews of ideal gas models [55], [56]. Most work has been based on simulations and there have been few field tests to address doubts regarding the non-random movements of real animals, their non-random dispersions, and their frequently non-independent movements (but see [53]).

In this paper, we separate animal movements into their day range and tortuosity components to examine the FMP formula. We use three levels that progressively decrease randomness and increase the realism of movements and space use (Table 1). If the FMP formula is fundamentally valid, specific shapes of animal movement paths should be irrelevant, i.e., a population of animals displaying linear movements and another population of equivalent density and day ranges but displaying convoluted movements would show no difference in their mean number of track crossings and would therefore be estimated with equal accuracy. We constructed these scenarios using virtual animal populations simulated to exhibit the desired parameters over the range of extremes expected to be encountered in real systems. We then examined two species that showed qualitative and quantifiable differences in the spatial patterns of their daily movements. Using accurate tracings of their actual daily travel paths, we simulated their populations with a random dispersion and tested how accurately the FMP formula could estimate their numbers. This same technique has also been employed previously with three species of deer and wild boar [52], [57]. Finally, there is an expressed need to compare FMP estimates of real populations with independent density estimates [52]. We make this comparison using two sympatric antelope populations since these animals are readily visible and amenable to distance sampling with line transects. Although we use examples from a specific context by necessity, our goal is broad and these explorations reveal a more general understanding of how animal movement parameters influence their detection. While some findings are not strictly novel, our purpose is to make these findings relevant and advance the field of tracking to benefit conservation.

**Table 1:** Differentiation of animal movements and dispersion with progressively increasing realism over three levels of testing.

	Animal spatial characteristics	
Level of testing	movement	dispersion
Simulation (virtual animals)	random	random
Simulation (empirical movements)	non-random	random
Real population comparison	non-random	non-random

## Methods

### Study Area

Data collection occurred in the KD1 Wildlife Management Area directly north of and adjacent to the Kgalagadi Transfrontier Park in southwestern Botswana. The Government of Botswana via the Ministry of Environment, Wildlife and Tourism and Department of Wildlife and National Parks granted approvals and permits (numbers EWT 8/36/4 XII (35), WP/RES/15/2/2 XXII (87)) to conduct the study within this publically owned, partially protected area. Since the field sampling techniques were non-invasive, ethics approval was not required. An area within 30 km of the unfenced park boundary was selected on the basis of its habitat uniformity and its high densities of the target antelope species. Human impacts in this area are minimal since the nearest settlement is a subsistence-pastoral community 70 km away. The country is relatively open semiarid savanna overlying a consistent sandy substrate. The plant community coincides with the *Schmidtia kalahariensis* type [58]; the dominant species are *Acacia luederitzii, Acacia erioloba*, *Grewia flava*, and *S. kalahariensis*. Visibility is good in the open savanna and tracking conditions are excellent. We collaborated with local tracking experts and horsemen from the adjacent remote area settlement of Zutshwa to conduct the field study.

### Track Counts

A single 10 km transect was created to bisect the unbounded study area. Track crossings were counted along this transect over six consecutive 24-hour periods by observers on specialised seats mounted to the front of a vehicle travelling at 6–8 km h^−1^. One expert local tracker and DK conducted all of the observations. No effort was made to eliminate subsequent crossings of the same individual animal. Surveys began at approximately the same time each morning (08∶00 h) and progressed at a similar rate, while concurrently a heavy steel beam was dragged behind the vehicle, which effectively obliterated tracks. This technique ensured a precise 24-hour period for track accumulation.

### Diel Animal Movement

We selected two ungulate species thought to exhibit general differences in both spatial dispersion and the pattern of their travel paths: gemsbok *Oryx gazella gazella* and steenbok *Raphicerus campestris*. We wanted accurate measures of these species’ daily travel distances and spatial tracings of their daily travel paths at high resolution.

We followed the tracks of individual animals to retrace the path that they walked. GPS data-loggers (Columbus V-900, Victory Technology, Fujian, China) programmed to take fixes at 1 s intervals captured fine-ruler tracings of each animal’s movement. Steenbok were tracked on foot and gemsbok were traced from horseback. Different ecologies dictated different approaches to obtaining diel tracings.

Steenbok pairs defend small territories (0.6 km^2^; [59]), which precludes forward-tracing their movements within a diel period because the presence of trackers invariably influences those movements. Instead, we opportunistically used rainfall events that reset the track record. When rainfall ended during the day, we sighted steenbok 24 h later. This was possible because steenbok are abundant and easy to see. From sighting, we back-tracked the animal to the point where the tracks became marked by raindrops.

For gemsbok, we spotted animals in the mid-morning. The next day, early in the morning, the animal was forward-tracked from the point of sighting. The tracing was terminated when the animal was re-sighted or when the animal obviously fled the approaching horsemen. In some instances, tracings were completed after 24 h had elapsed. Excess distance was subtracted from the travel record according to the fraction of the 24-hour period that had elapsed.

We used a simple metric of tortuosity, calculated as a ratio of the total daily travel distance divided by the distance between the start and finish locations, to quantify differences in spatial patterns of steenbok and gemsbok travel paths.

### Line Transects

Since both steenbok and gemsbok are abundant enough to be readily visible, we used distance sampling with line transects to independently estimate density. We sampled along three parallel, equally spaced 10 km transects, each separated by 3 km. The centre transect was the same as that used for the track counts. Transects were created simply by driving a vehicle off-road and were sampled several times during daylight hours at a speed of 20–30 km h^−1^. Animals were spotted by the driver and by two observers positioned on the tracker seats. When animals were spotted, their group size was determined and the vehicle was stopped when the line of sight to the animal(s) was at an angle perpendicular to the transect. The distance between the animal(s) and the transect was determined with a laser rangefinder. Occasionally, when animals fled before the vehicle could reach the perpendicular location, a tracker would walk to the place where the animal(s) was standing so that an accurate reading could be obtained with the rangefinder. Densities with 95% bootstrap confidence intervals (CIs) were estimated using conventional distance sampling [60] with Distance 6 software [61]. We selected detection probability functions and adjustments based on Akaike Information Criterion and graphical best fits to the sighting data.

### Simulations

We simulated virtual animal populations exhibiting incremental levels of travel path tortuosity (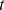), across combinations of density (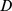) and day range (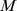) expected to approximate the range in variation of most terrestrial species for which tracking is applicable.

We began with a conceptual area of 2500 km^2^ (50×50 km^2^). For each scenario of animal 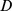, 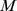, and 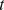, one straight-line transect 10 km in length was imported into the area with a random starting location and orientation. Then, using an appropriate density, “animals” were randomly imported as points from which they moved in random directions to the specified 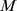 and 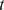, as described below. This process was repeated 1200 times, resulting in a 12000 km survey effort for each permutation of 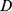, 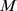, and 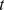. We simulated 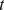 by beginning with a population exhibiting straight-line movements, then incrementally increased the number of “turns” the animals made by breaking the movement paths at random distances and assigning a random turn angle at each vertex. This approach simulates an uncorrelated or pure random walk. Incremental tortuosity was denoted by 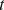 = 0 (straight lines), 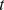 = 1 (single turn), 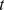 = 2 (two turns)… 10, 20, 30, 40, 50. Within each level, the tortuosity of individual “animal” paths varied widely because the turn angles were random (between 0 and 2π); however, the average tortuosity for the population increased in proportion with the total number of turns. The levels of movement length were 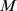 = 0.3, 3, 10, 30 km and the levels of density were 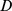 = 0.0004 (one animal), 0.0002, 0.004, 0.002 0.04, 0.02, 0.4, 2, and 4 km^−2^. Intersections between both every “animal” travel path and between each path segment and the transect were summed for each transect.

To increase the spatial realism of the simulation, virtual populations were unbounded by the conceptual area. Animals were dispersed randomly at a specified density within the area, but equally throughout a larger buffer area. The animals were then permitted to move without regard to boundaries. Transect intersections included animals originating inside and outside the conceptual area. For each scenario, an equal number of animals were just as likely to move from inside the area to outside the area and vice versa. Structuring the simulation in this way avoided edge effects and most closely approximated reality when applying a track transect survey to an unenclosed population.

In addition to virtual populations, we simulated populations of both antelope species using their real travel paths. Empirical paths were pulled randomly with replacement from the available data set and imported into the conceptual survey and buffer area with random starting points and orientations until the desired number of animals was reached for a range of densities from 0.02–4 km^−2^. A 10 km transect was then imported with a random starting point and orientation, over which the transect intersections were enumerated. This process was repeated 500 times. Notably, the locations and orientations of both the travel paths and transects were randomized over each iteration. The same consideration for movement in and out of the study area was also applied.

### FMP Calculations

We used nonparametric bootstrapping [62] to calculate the uncertainty in the FMP density estimates. For real populations, both daily replicate transects and available movement paths were resampled with replacement at original sample sizes to produce bootstrap replicates of 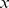/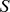 and 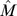, from which one estimate of 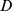 was calculated using the FMP formula. This process was repeated 5000 times to generate the distribution of 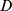 for each species, from which the mean and bias corrected and accelerated 95% CIs were calculated. We used a similar approach for the simulated populations, whereby bootstrap replicates of 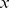/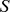 were generated by resampling from the entire data set of iterations in proportion to the appropriate survey effort (i.e., 100 km survey effort generated by resampling 10 random transect iterations, 250 km from 25 transects, etc.). The calculations for virtual populations differed only in that *M* was a single value and therefore did not contribute to uncertainty in the resulting density estimates.

## Results

### Simulated Animal Movements

The fundamental linear relationships defined by the FMP formula were verified by the simulation results. For example, a doubling of 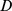 results in a doubling of 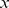/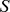, which corroborates previous findings [57]. Similarly, it was clear that for a constant value of 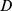, a doubling in 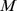 results in a doubling of 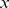/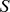.

**Figure 1:**
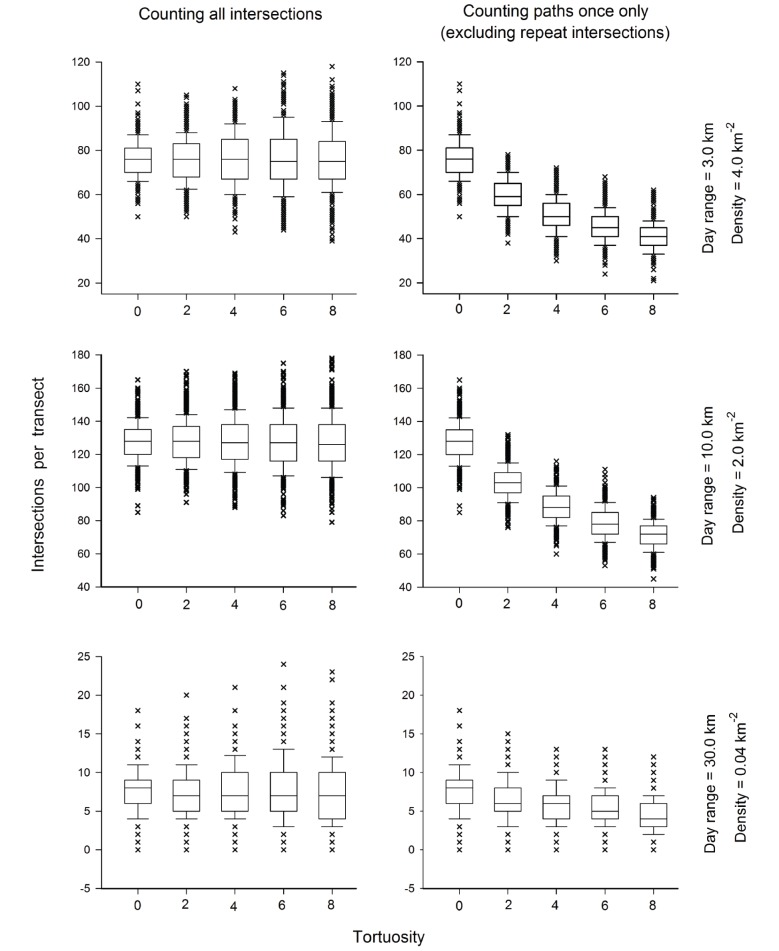
Sample output from three combinations of simulated daily travel paths and densities. Box plots with outliers are shown; each data point represents the numbers of intersections per transect (500 iterations) across five arbitrary levels of travel path tortuosity.

When 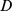
and 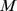 were held constant, the mean number of intersections per transect did not change over levels of 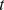, from straight-line movements to highly tortuous random walks. A subset of outputs from several combinations reveals this consistency (left panels in Fig. 1). Because the mean encounter rates did not change, the FMP formula estimated densities accurately regardless of the shape taken by the travel paths. At the maximum number of transect replicates (1200), the mean estimates from all scenario combinations deviated by a maximum of 2% from the true simulated densities.

**Figure 2:**
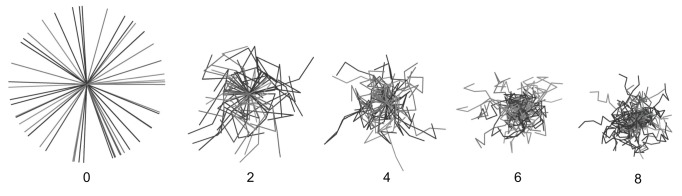
Displacement of simulated animal travel paths over levels of tortuosity. Fifty travel paths of equal length originate from a common centroid for each level of tortuosity (numerals indicate the number of random turn angles).

Although tortuosity had no effect on the mean encounter rates and the subsequent accuracy of the FMP estimation, detectability was affected. Detection probabilities, reflected by the number of individual animals that intersected transects, declined with increasing tortuosity (right panels in Fig. 1). Simulated animal movement paths originating from the same point (Fig. 2) help to visualise the declining detectability that resulted in the pattern in Fig. 1 (right panels). With increasing tortuosity, the average displacement covered by the paths decreased, so that paths at t = 8 covered just over half of the Euclidean distance as t = 0. Transects that sampled populations exhibiting the most tortuous paths (t = 50) counted fewer than 30% of the individual animals that were counted when those populations exhibited straight-line movements (t = 0). The results indicated that detectability is determined by both day range and tortuosity. This effect could only be established via simulations because in a majority of situations it is impractical and impossible to determine with certainty if tracks belong to the same or different individual animals.

**Figure 3:**
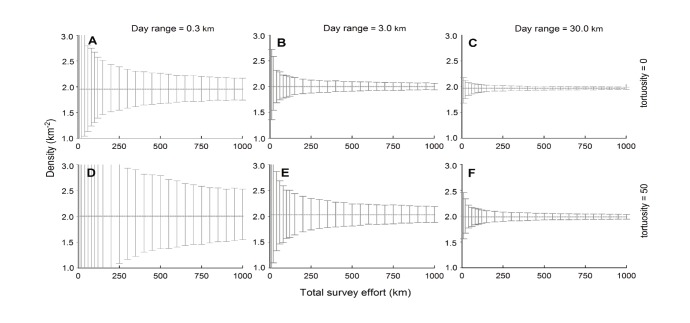
Effect of daily travel distance (column panels) and path tortuosity (row panels) on FMP estimate precision. Mean densities and 95% CIs are shown from applying the FMP formula to 10 km transects sampling virtual populations at 2 km^−2^. Dotted lines indicate the accuracy of mean density estimates at 1200 replicates, which vary within 2% of the true density. Note that both day range and tortuosity influenced achievable precision.

A further consequence of the interaction of day range and tortuosity is uncertainty in the resulting density estimates. The sample variance increased when the travel paths became shorter and more convoluted. The effect is apparent over a broad range of expected daily movements for terrestrial species (Fig. 3). Species with smaller bodies are more likely to occupy the low end of day range (0.3 km); examples include tortoises, some weasels, mongooses, primates, and likely many rodents [63]–[65]. At the other extreme, spotted *Crocuta crocuta* and brown hyaenas *Hyaena brunnea* in the Kalahari have been recorded moving on average 26.5 and 31.1 km per night, respectively [66]. However, the majority of terrestrial species for which track counts are applicable are likely to have daily ranges somewhere in between these values (see [64], [65]). When the survey effort reached 250 km (1 km sampled for every 10 km^−2^), the 95% CIs ranged at the extremes from 54–154% of the true density (panel d of Fig. 3) to 97–102% of the true density (panel c of Fig. 3). However, these results likely overestimate the precision that can be achieved in real populations because the virtual animals in the simulations were dispersed randomly, the group size was therefore one animal, and 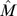 did not vary. Therefore, the outputs in Fig. 3 primarily illustrate the general effect of day range and tortuosity on precision.

### Simulation Using Empirical Travel Paths

We traced 17 gemsbok and six steenbok diel travel paths. Despite body sizes that differ by over an order of magnitude, the two species’ daily movement distances did not differ considerably; gemsbok travelled 5.65 (coefficient of variation 0.42) km on average and steenbok travelled 4.20 (0.34) km on average. However, the patterns of their travel paths were markedly different. Gemsbok had more linear movements, covering larger areas in the landscape. This aspect was reflected in a tortuosity metric of 4.22 (0.62). Steenbok, confined to relatively small territories, displayed much more tortuous movement patterns, with a tortuosity metric of 10.86 (0.31).

**Figure 4:**
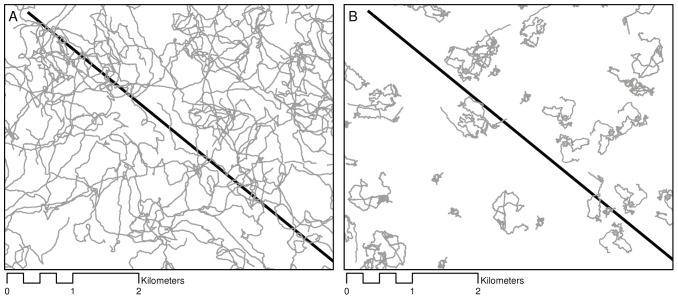
Empirical daily movements dispersed randomly in simulation space. Image capture (1∶50 000) shows a single iteration of simulation runs at 2 km^−2^ density for (A) gemsbok and (B) steenbok. Approximately half of the randomly oriented transect (black) appears diagonally, underlying travel paths (grey). Note that both gemsbok and steenbok have similar daily travel distances but display different tortuosity in their movements, resulting in different spatial use.

When empirical movements were dispersed randomly in the simulation space, gemsbok had higher detectability than steenbok by virtue of the differences in the shapes of their travel paths and resultant space use (Fig. 4). Considering day ranges that differed by only 34.5%, at equivalent densities, 3.3 times more individual gemsbok were detected than steenbok per transect on average. However, if a gemsbok was detected, it was likely to intersect a transect 2.2 times on average. In contrast, if a steenbok was detected, it was likely to intersect a transect 5.4 times on average.

**Figure 5:**
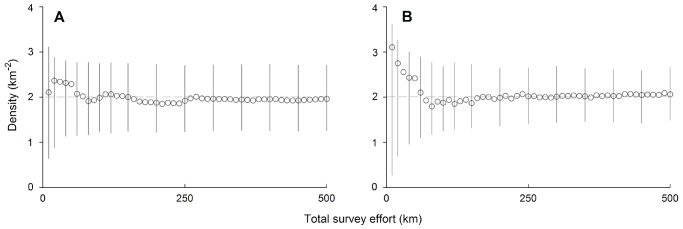
Estimates from simulated densities (2 km^−2^) using empirical movements of (A) gemsbok and (B) steenbok. FMP point estimates of density from a random cumulative increase in survey effort (10 km transects) are displayed along with 95% CIs.

Over the range of simulated densities, when transects were replicated 500 times, the FMP formula returned mean estimates within 5% of their true value, which is further evidence that the estimator is unbiased by the specific shapes of animal movement paths. For example, when the population density was 2 km^−2^, the number of gemsbok was estimated to be 1.97 km^−2^ and the number of steenbok was estimated to be 2.03 km^−2^ (Fig. 5). The accuracy of these mean estimates approached the true densities once the cumulative survey effort reached about 250 km or a sample penetration [44] of 1 km of transect per 10 km^2^ of survey area. At this effort, CIs around point estimates were 73% of the mean density for gemsbok and 54% of the mean density for steenbok. This precision was poorer than that of deer from Stephens *et al*. [52] due to less precise estimates of 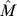
arising from smaller sample sizes. The effect of variation in 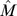 on the precision of the density estimates is illustrated by comparing with virtual populations where the day range was constant (see the spread of 95% CIs in Fig. 3 versus Fig. 5).

### Real Population Comparison

**Figure 6:**
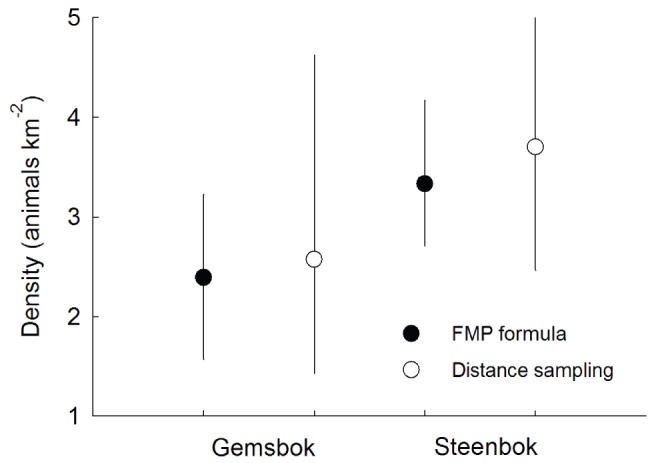
Density estimates of two empirical populations using the FMP formula and Distance sampling. Displayed with 95% bootstrap CIs.

Both antelope species had similar encounter rates along the track transect: gemsbok with 8.59 intersections km^−1^ 24 h^−1^ on average and steenbok with 9.58 intersections km^−1^ 24 h^−1^. Combining these data with their respective day ranges in the FMP formula returned density estimates for gemsbok (2.39 km^−2^; 95% CI: 1.57–3.23 km^−2^), and steenbok (3.33 km^−2^; CI: 2.71–4.17 km^−2^). Line transects (394 km) revealed 74 gemsbok observations (270 individuals) and 66 steenbok observations (72 individuals). Conventional distance sampling analyses and bootstrap CIs produced estimates for gemsbok (2.57 km^−2^; CI: 1.43–4.62 km^−2^), and steenbok (3.7 km^−2^; CI: 2.47–5.55 km^−2^). Despite small sample sizes and unknown true densities, the two independent approaches returned density estimates that were closely matched (Fig. 6). Assuming that the distance-based estimates are accurate, this limited comparison is suggestive that the FMP estimator was also accurate and robust to non-independent animal movement patterns and non-random dispersion.

Track-based estimates were more precise than distance-based estimates (Fig. 6). Transects were only 10 km in length, so direct observations of animals per transect were limited and several line transects had zero counts for each target species. As a result, it was necessary to sample two additional line transects in parallel to the centre transect to obtain a minimum number of sightings for estimating detection functions. In contrast, track counts captured close to 100 observations per transect. There was higher variance in the numbers of observations on different line transects (CVs of 0.96 and 0.84) compared with track transects (CVs of 0.42 and 0.11) for gemsbok and steenbok, respectively, which was reflected in the wider CIs shown by the distance-based estimates compared with the FMP estimates.

## Discussion

When it is suggested that counts of animal tracks can be used to estimate population density a remarkably immediate and consistent question from both biologists and laymen is “but how do you avoid over-counting the same individual animals?” This issue seems intuitively problematic. Repeated counting of individual animals’ tracks along a transect or between spatial replicates during a survey is frequently viewed as a problem. Some efforts have attempted to reduce the rate of re-counting individual animals by using arbitrary exclusion distances between sets of tracks [67], [68] or by separating transects sufficiently in space so that the probability of a single animal being recorded on more than one line is minimized [39], [25], [69], [70]. Reliably distinguishing individuals based on their tracks is much more difficult and perhaps possible among a few species such as large cats [71]–[74], rhinos [75], tapirs [76], and potentially elephants [77]. However, exceptional trackers or detailed measurements and sophisticated analyses are required. In contrast, counting every track intersection is repeatable and simpler than attempting to separate individual animals, but rarely implemented because such counts are considered to be difficult to interpret [78]. At the least, track surveyors typically make some effort to eliminate obvious re-crossings that are visually connected [52], [79]. Decisions must be made at the outset of every program whether to discount re-crossings of same individual animals, simply record presence over some spatial dimension, or enumerate each and every track. The literature reflects little agreement on an optimal approach.

If density estimates are sought, the FMP formula suggests that re-counting the same individual animals is not a problem and that it is in fact desirable to count the same individuals if they re-cross transects within the same 24-hour period, as many times as they do. Geometry dictates a balance between the number of intersections and the length of line segments, regardless of the shapes of the lines. The inference is simply that individuals with more tortuous movements are detected less but, when encountered, those individuals are generally counted a larger number of times by virtue of the convoluted pattern of their movement. Detectability is influenced by tortuosity; the total number of intersections is not. The FMP formula describes the relationship between counts and true density if correct track counting rules are applied. A strict definition of detectability includes the probability that tracks are observed after they intersect a transect. We expect this probability to approach 1 in the Kalahari, where tracks are easily visible and can be verified by more than one expert observer. However, surveyors in different parts of the world surely have wide variation in tracking skill level (see [71], [80]) and tracker proficiency should be addressed more often [81]. Nonetheless, our consideration of detectability here has been limited to the more fundamental probability of intersection between animals and transects. This detection probability remains an imprecise concept, determined by the interaction of day range and path tortuosity. Among two populations with equal movement rates, we have shown that those with more tortuous movements have lower detectability. Likewise, if two animals have equally tortuous movements, the animal with a longer day range will have higher probability of being detected. The interaction of these two travel path parameters can perhaps be conceptualised as the displacement that animals cover during their daily patterns of movement, i.e., those individuals that cover larger distances in Euclidean terms have greater detectability.

### Implications for Occupancy

Track surveys have often been applied to model the fraction of sampling units in a landscape where a target species is present (occupancy) in order to monitor distributional changes [68], but also as a surrogate for abundance to monitor trends in population sizes [82]–[84]. Animals have high detection probabilities by their tracks because such indirect observations are time integrated and reflect animal presence over an area typically much greater than the space within which animals can be observed directly at a particular moment. For example, 95% of gemsbok and steenbok sightings along line transects in the present study occurred within 355 and 120 m, respectively. Track counts certainly captured animals that had travelled from, or to, a substantial distance beyond which direct sightings are possible. This factor contributed greatly to track observations in the 8–10 km^−1^ range, while some line transects failed to detect either species.

Minimizing the imperfect detection of species (false absences) has become a key concern of occupancy studies [85]–[87]. Although the FMP formula is unaffected by the vagaries of specific spatial patterns of animal movements, applications utilising presence-absence data from indirect sign are vulnerable to biases emerging from changing animal detectability. For example, when empirical movement paths were imported randomly to a density of 0.04 km^−2^ (100 animals within the study area), a survey effort of 100 km (10 transects) had a >99.9% probability of detecting gemsbok presence, but an 86% probability of detecting steenbok presence in the area. When 500 transects were applied to these populations in a single survey, 51% of individual transects detected gemsbok, while the presence of steenbok was recorded on only 18.2% of transects. Differences in detectability between these two species due to tortuosity can be seen in Fig. 4. The tortuosity of animal movement paths may fluctuate widely within species and individuals for any number of reasons that are difficult to predict [88]. Since detection probabilities of animals by their tracks are not constant, even over short periods (day to day), an appropriate occupancy design would require repeated sampling and assume no unmodelled heterogeneity in detection to make reliable inferences (see [89]). The key concern is whether these heterogeneous detection probabilities can be captured adequately by a combination of environmental covariates and conditions specific to track accumulation period [89], or by extending the interval for track accumulation over several days [70].

It is often reiterated that occupancy studies are advantageous because presence-absence data are often easier and less expensive to collect than count data (e.g., [86], [90]–[93]). However, this suggestion is doubtful in the case of animal track surveys. Since all animal tracks have to be observed during a survey, we suggest that little additional effort is required to count every track intersection, from which presence-absence data are easily extracted later, if desired. Hayward et al. [29] reported that despite increased variance caused by counting repeat track intersections along transects, this index had more power to detect declines in Amur tigers *Panthera tigris altaica* than did presence-absence data. Presence-absence studies frequently report low power and capability to detect only large trends [20], [82], [94], [95], require intensive sampling protocols with a large number of replicates and repeated sampling over short periods [85], [89], and necessitate restrictive assumptions regarding independence of sampling units [70], [96]. In contrast, the FMP estimator embraces count data while dispensing with concern over individual animals being detected in more than one sampling unit and negating the explicit requirement to estimate detectability. In many cases, the FMP formula may provide a more parsimonious approach than modelling occupancy as a surrogate for indexing abundance and monitoring population trends from animal tracks.

### Implications for Indexing

FMP theory clarifies the implicit assumption of all efforts that use track counts as indices of relative abundance with which to monitor change: average daily travel distances remain constant. This fact of course applies equally to the indexing of camera trap rates to density [97], [98]. Practitioners need to appraise the extent to which this assumption is true for populations separated in time or space. If day range is density dependent, the assumed monotonic linear relationship between track counts and true density will not hold. For example, it is possible that a drop in density with declining food availability may be coupled to a disproportionate increase in day range as animals expand their home ranges or disperse [99], [100]. Changes may occur over relatively short periods. For example, in applying the FMP formula to estimate deer densities, Stephens et al. [52] subdivided movement data due to differences in day range between early and late winter. Irrespective of whether track counts or camera trap rates are used as relative indices or converted to density using the FMP formula and other random encounter models, there are obvious implications for the frequency with which day range needs to be reassessed when monitoring populations.

Calibrating track indices to independent estimates of true density, then applying those linear models to estimate density in other areas, is a growing practice applied to large carnivores in southern Africa [45]–[47], [101]–[103]. It is assumed during data collection that individual animals can be differentiated and counted once only during a survey, which may be closely approximated with the help of extremely skilled trackers [71]. Stander [44] first mentioned “range utilisation,” “habitat use,” and “behaviour of species” influencing the slope of the linear relationship between track counts and true density. If individual animals are recorded only once during a survey (and subsequent re-crossings are ignored), then the present results confirm that the shapes of those individual travel paths will become important in the index–density relationship. Stander’s [44] comments are valid since stable animal path tortuosity must be assumed, including the assumption that movement parameters of the populations used to generate the linear calibration model do not differ from the populations to which the calibration model is applied. Furthermore, when multiple species are combined into a single linear model [47], this assumption must be extended to: all species used to generate the model and to which it is applied have equal day ranges and movement path tortuosities.

Large carnivores in particular pose a challenge to FMP application because their low densities require large survey efforts, and the logistical practicalities of large survey efforts often dictate convenience sampling by vehicle along pre-existing linear features. Some species such as brown hyaenas are quintessential trail users and most large carnivores habitually use linear features for ease of travel. Indeed, many indexing and occupancy approaches are based on such behaviour [24], [82], [84], [104]. Recent studies [53], [105] have highlighted the importance of random placement of camera traps with respect to naturally non-random animal movements to avoid biased inferences – a warning that applies equally to track transects and the FMP formula. Even though predators disproportionately utilise roads and trails throughout a landscape, randomly located sampling points or transects with respect to these linear features will return unbiased estimates at the landscape scale [54]. In contrast, applying the FMP formula to large carnivore-specific surveys whereby transects are situated non-randomly along convenience features [45]–[47], [101]–[103] would presumably result in biased density estimates. In a practical sense, it would be useful to know whether these bias errors are generally larger or smaller than the bias errors resulting from collapsing differential day ranges of multiple species into a single index calibration model [47], fluctuations in both day range and tortuosity in the animals to which the calibration model is applied, and the error involved in isolating individuals by their tracks. Sampling along roads and trails is always more practical, especially when large survey efforts are required, but practitioners should strive for random transects with respect to animal movements for unbiased inferences when applying the FMP method.

## Conclusions

Our attempts to disprove the FMP formula through both virtual and empirical tests revealed no flaw in the simple equation. It appears that the number of animal crossings along lines depends simply on the density of those animals and how far they walk; the shape of specific movement paths is irrelevant. While spatial elements of animal movements have no fundamental bearing on accuracy, biases may arise from the placement of transects with respect to the distribution of animals and principles of good survey design, such as appropriate stratification, apply to any method used to survey biological populations. We also stress that the sampling intensity and total survey effort required to achieve desirable levels of accuracy and precision in density estimates will depend on dispersion, day range, and movement patterns, in addition to density and group size [52], [57]. In particular, populations with lower density, clumped dispersion, larger group sizes, shorter daily movement distances, and greater tortuosity will require larger survey efforts to achieve the desired accuracy and precision. The main practical limitation to the FMP approach is obtaining accurate estimates of day range. While our capacity to obtain and share animal movement data continues to grow with advances in GPS technology, our ability to estimate day range accurately from these data remains presently limited [106]. However, even coarse estimates of day range can be profitably applied to the FMP formula for many species whose abundances are impossible to estimate by other means [107].

Bearing the above in mind, the FMP formula should be applicable to any terrestrial species with readily observable tracks if three assumptions are met: (1) animal movements are random with respect to transects, that is, naturally non-random animal movements are not influenced by the presence of a transect, (2) all animals that intersect transects are detected and identified correctly, and (3) all intersections are enumerated regardless of individuals. Several track-based research and monitoring programs use methods that already accommodate these assumptions, including long-term data sets in the northern hemisphere [6], and many more could easily be made amenable. Russian biologists have understood and have been using the FMP formula for decades. It is fortunate that this formula has become available to English speakers because conservation practitioners around the world can benefit from understanding and utilizing the FMP formula.
